# P-887. Time for change: Cefazolin prophylaxis is safe for penicillin allergic patients undergoing joint arthroplasty, a single centre experience

**DOI:** 10.1093/ofid/ofaf695.1095

**Published:** 2026-01-11

**Authors:** Trevina Lee, Chong Hua Kang, Peijun Yvonne Zhou, YiBo Wang, Haur Yueh Lee, Darren Keng Jin Tay, Maciej Piotr Chlebicki, Shimin Jasmine Chung

**Affiliations:** Singapore General Hospital, Singapore, Not Applicable, Singapore; Singapore General Hospital, Singapore, Not Applicable, Singapore; Singapore General Hospital, Singapore, Not Applicable, Singapore; Singapore General Hospital, Singapore, Not Applicable, Singapore; Singapore General Hospital, Singapore, Not Applicable, Singapore; Singapore General Hospital, Singapore, Not Applicable, Singapore; Singapore General Hospital, Singapore, Not Applicable, Singapore; Singapore General Hospital, Singapore, Not Applicable, Singapore

## Abstract

**Background:**

Non-ß-lactam antibiotics are often used for surgical prophylaxis in penicillin allergic (PAL) patients due to fear for adverse events. However, it is not without risk. The avoidance of cefazolin (CFZ) prophylaxis has been shown to increase surgical site infection infections (SSIs), *C. difficile* infection and antibiotic resistance. It is increasingly known that there is low risk of cross reactivity between penicillin and CFZ, prompting some orthopaedic surgeons to switch to CFZ prophylaxis. We aim to evaluate the safety and efficacy of CFZ in PAL patients undergoing joint arthroplasty at our centre.

**Table 1:**
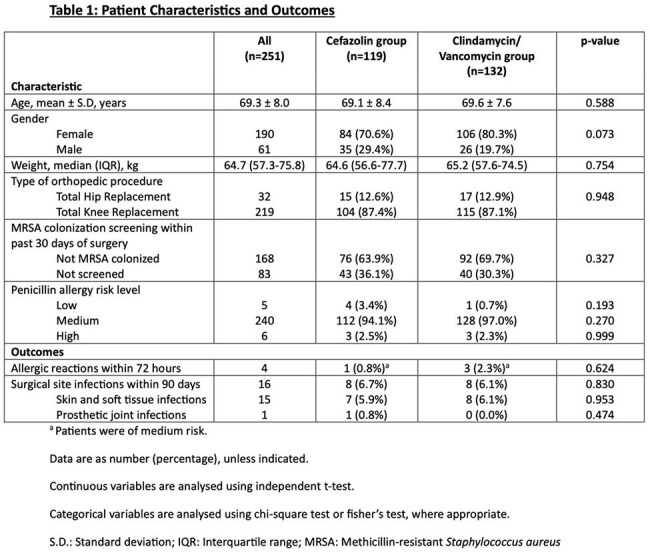
Patient Characteristics and Outcomes

**Methods:**

This is a single centre, retrospective, observational study performed as a clinical audit comparing PAL patients ( > 21 years old) receiving CFZ versus clindamycin/ vancomycin (CV) for joint arthroplasty (hip/ knee) at Singapore General Hospital (SGH), between Jan 2023 to Jun 2024. PAL patients with severe cutaneous adverse reaction were excluded. PAL patients were further classified to low (adverse effects without cutaneous/ cardiorespiratory involvement), medium (rash/ angioedema without systemic effects), and high (cardiorespiratory involvement) risk. Surgical prophylaxis was continued for 24 hours as per institution guidelines. The incidence of any reported allergies within 72 hours and SSIs within 90 days of antibiotic use between the 2 groups were reviewed.

**Results:**

A total of 251 patients were included; 119 (47.4%) and 132 (52.6%) patients received CFZ and CV respectively; most were of low/medium risk (245/251, 98%). Possible mild allergic reaction occurred in 1/119 (0.8%) and 3/132 (2.3%) patients from the CFZ (itch) and CV groups (rash in 2 patients, itch in 1 patient) respectively (p= 0.624). All 4 patients were of medium risk.

Post-operative SSIs occurred in 8/119 (6.7%) and 8/132 (6.0%) among CFZ and CV patients respectively (p= 0.830).

**Conclusion:**

CFZ prophylaxis is safe in PAL patients. It is imperative for antibiotic stewardship teams to drive behavioural changes among prescribers to encourage accurate allergy assessment and appropriate CFZ prophylaxis where appropriate. Prescriber education on PAL must be reinforced, and implementation of risk-stratified institution guidelines may be considered.

**Disclosures:**

All Authors: No reported disclosures

